# Remsima (a Tumor Necrosis Factor (TNF) -α Inhibitor) induced hemolysis in a patient with Crohn's disease - Case report

**DOI:** 10.1016/j.amsu.2021.102768

**Published:** 2021-08-25

**Authors:** Rehab Y. AL-Ansari, Arwa AL. Khuraim, Leena Abdalla, Hind Hamid, N.Y. Zakary

**Affiliations:** a Adult Hematology Unit, Internal Medicine Department, KFMMC, Dhahran, 31932, Saudi Arabia; b Internal Medicine Department, KFMMC, Dhahran, 31932, Saudi Arabia; c Gastroenterology Unit, Internal Medicine Department, KFMMC, Dhahran, 31932, Saudi Arabia

**Keywords:** Hemolytic anemia, Infliximab, Drug induced hemolysis, Crohn's disease, Tumor necrosis factor inhibitor (TNF) -α, Remsima

## Abstract

**Introduction:**

Crohn's disease (CD) is an idiopathic inflammatory disorder of unknown etiology with genetic, immunologic, and environmental influences. Infliximab is a treatment modality for fistulated Crohn's disease. Infliximab induced hemolysis is rare and very few cases reported before in Ulcerative colitis (UC) but not in Crohn's disease**.**

**Case presentation:**

We are reporting a 63 years old gentleman who was diagnosed as Crohn's disease and started on Tumor necrosis factor Inhibitor (TNF) -α i.e. (infliximab - Remsima) infusion. The course was complicated by Coomb's negative hemolytic anemia which is suggestive of non-immune drug induced hemolysis. Our patient was treated with steroid and conservative measures. Upon following up, his hemoglobin level as well as all hemolytic markers showed dramatic improvement. Adalimumab was used to treat this patient as an alternative choice without relapse of hemolysis.

**Clinical discussion:**

Drug induced Hemolysis is not a well-known complication post receiving Tumor necrosis factor Inhibitor (TNF) -α infusion in patients with Crohn's disease. Coombs negative hemolysis keeps in favor of non-immune drug induced rather than other differentials in our case scenario.

**Conclusion:**

Although cross-reactivity is expected between one biological agent to another, in our case the use of Adalimumab as alternative choice post Tumor necrosis factor Inhibitor (TNF) -α (Remsima infliximab) induced hemolysis did not cause hemolysis or any type of reaction.

## Highlights

• Crohn's disease is one of the Inflammatory bowel diseases (IBD) that can affect any part of gastrointestinal tract as well as extraintestinal organs.
• Tumor necrosis factor Inhibitor (TNF) -α i.e. (infliximab - Remsima) is a treatment modality for fistulated Crohn's disease.
• Drug induced Hemolysis is one of the complications that is not well-known post receiving Tumor necrosis factor Inhibitor (TNF) -α (Remsima) infusion.

## 1 Introduction

Crohn's disease is one of the Inflammatory bowel diseases (IBD) that can affect any part of gastrointestinal tract as well as extraintestinal organs and could end with serious complications such as anal fissure, intestinal stricture and fistula formation [1]. Tumor necrosis factor Inhibitor (TNF) -α as Infliximab is an effective treatment for Crohn's disease [2]. In study by Hamzaoglu H et al., serious short as well as long-term adverse events can be seen in up to 15% of patients who are receiving Infliximab and the main side effects were sepsis, serum sickness, lupus induced and malignancy [3]. Hematological adverse events such as pancytopenia, lymphoma, hematological malignancy and autoimmune hemolysis were reported with Infliximab infusion in inflammatory bowel disease mainly with Ulcerative Colitis [4]. Tumor necrosis factor Inhibitor (TNF) -α induced Coomb's negative hemolysis as in our case scenario, can be one of the rare complications after exposure to Remsima which is worth reporting. This article has been reported in with the SCARE criteria [5].

## 2 Case presentation

This is a 63-years-old male known to have hypertension on Amlodipine 5 mg per oral daily. Has no background of any hematological disease. He was initially presented to our center with a picture of subacute small bowel obstruction evident clinically and radiologically which improved with conservative management. As part of evaluation both endoscopy and colonoscopy were done which showed mild gastritis and normal large bowel up to the cecum as the terminal ileum couldn't be reached. Further Investigations were requested, p-ANCA, ANA, ASCA, Fecal calprotectin, TB QuantiFERON and CT enterography was requested. ASCA came to be positive and ANA with p-ANCA were positive as well, Fecal calprotectin 191, H. Pylori was positive. CT enterography showed inflammatory changes highly suggestive of Crohn's disease.

As the patient has active disease flaring and TB Quantiferon result was negative, he was started on Prednisolone 30 mg per oral daily with tapering dose and Infliximab (Remsima). Two doses of infliximab (Remsima) were given 2–3 weeks apart. After the second dose of Remsima and during admission for the third dose, the patient gave history of change of the color of the sclera to yellow color and active dark cola colored urine which started two weeks prior to current admission along with progressive anemia symptoms in form of dizziness, fatigability, transient palpitation and occasional headache for ten days duration. The third dose of Remsima was held, and ultrasound abdomen with new hematology and chemistry lab work was requested.

**Fig. 1:**
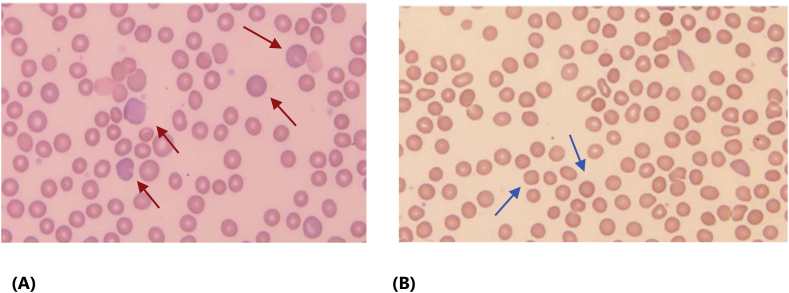
Peripheral Blood Film Showed **(A)** Polychromatic Red Blood Cells and Reticulocytes (red arrows) **(B)** Full of Red Blood Cells Spherocytes (blue arrows) with occasional fragmented cells. (For interpretation of the references to color in this figure legend, the reader is referred to the Web version of this article.)

Laboratory results showed dropping in Hemoglobin from 13.9 to 7.16 g/dl which was confirmed by repeating CBC samples. Moreover, reticulocyte 5.4%, Haptoglobin <0.308, Coomb's test was negative, high LDH, AST and Total Bilirubin, laboratory picture goes with hemolytic anemia (Tables 1A and 1B). Peripheral blood film showed a picture of Polychromasia with increased Reticulocytes, Macrocytic red blood cell with Occasional nucleated red blood cells seen but no Schistocytes or blast cells (Fig. 1 A and 1B). Ultrasound abdomen excluded biliary obstruction.

Additional test as repeated ANA came to be positive, Anti double strand DNA IgG was negative, C3 and C4 complements were normal (1.49 and 0.382 g/l respectively). G6PD secreening was normal.

### 2.1 Physical examination

A well-built black male fully oriented and conscious, not in any distress and has an icteric sclera. His vital signs were stable and afebrile with a temperature of 37.2C. There were no palpable cervicale, axillary or groin lymphadenopathy. Insignificant respiratory, cardiovascular and gastrointestinal examination. Musculoskeletal and skin examination were unremarkable.

### 2.2 Management and follow up

Given the hematological laboratory findings with his direct Coombs test negative result, hemolytic anemia was diagnosed, and it was concluded that his anemia was drug-induced hemolysis from Tumor necrosis factor Inhibitor (TNF) -α (Remsima) infusion. A decision was made to discontinue Remsima treatment and monitor serial hemoglobin levels and hematocrit. One unit packed red blood cells was transfused aiming for a hemoglobin level of above 8 g per deciliter. Empirical course of a vitamin B12 injection with folic acid 5 mg per oral daily along with iron supplements was given. Adding to that, the patient was kept on Prednisone 1mg/Kg/day for one week followed by a slow tapering course over six weeks. Due to the unavailability of other lines of management, Adalimumab as an alternative choice was considered to be started four to six weeks post hemolytic event. The Patient was discharged from the hospital after 48 hours observations with stable hemoglobin of 8.5 g/dl for two readings. The patient was reevaluated in outpatient unit two, four and six-weeks post discharge during which tapering down of prednisolone dose was ongoing and all hemolytic markers showed improvement if not normalization (Table 1A, Table 1BA and 1B). Adalimumab loading dose followed by 40 mg subcutaneous injection alternating weeks doses which was started three months post hemolytic event with no further hemolysis. Moreover, as Patient has repeated positive ANA pre and post Remsima therapy, in addition to previous positive P- ANCA referral to rheumatologist was done for further workup.

## 3 Clinical Discussion

Hemolytic anemia is a condition where red blood cells get destroyed earlier than normal. It is classified into three main categories, either autoimmune, alloimmune or drug induced hemolysis [6]. Drug induced hemolysis accounts for 10% of acquired hemolytic anemia [7]. Acetaminophen, cephalexin, ceftriaxone, penicillin, ampicillin, chlorpromazine, ibuprofen, interferon alpha, procainamide, quinidine and rifampin among the most frequent medications that can cause drug induced hemolysis. Coomb's test is a useful tool as guidance for the type of hemolysis that we are dealing with. It will be positive in drug induced immune hemolysis as in penicillin but can be negative in drug induced none immune hemolysis as in ribavirin due to direct destruction of red blood cells (RBC) without immune mediated activity.

Warm autoimmune hemolytic anemia (AIHA) can occur in 0·5–0·7% of patients with ulcerative colitis (UC) but it is rare in Crohn disease (CD). The treatment of AIHA in IBD is by steroids in addition to immunosuppressive agents such as azathioprine and exceeding that to infliximab or cyclosporin in uncontrolled AIHA secondary to disease activity [7]. Autoimmune hemolysis secondary to infliximab infusion in case of Ulcerative colitis was reported by Fazia A. Mir et al., Vermeire S et al. and Fidder H et al. [[8], [9], [10]]. One reported case was published by Bong Soo Park et al. for Coomb's negative AIHA in case of a CD who was on Mesalazine but no report about any other drug precipitated the condition [11].

Debate whether this case is lupus induced by Infliximab infusion or drug induced hemolysis was there. Jason B Klapman et al. reported a case of lupus like syndrome post Infliximab therapy by almost eight months that was manifested by increasing in ANA, Anti DNA and low C4 with clinical picture of lupus manifested by arthritis, lupus urticarial and papulo-squamous rash. The event in that case required interrupted courses of the steroid and methotrexate in addition to stopping the Infliximab [12]. In our case scenario the patient already has the grave a positive ANA and p-ANCA prior to use of Infliximab. Furthermore, there was no clearance or normalization of the ANA post stopping the Infliximab as in Jason B Klapman reported case even though ANA post Infliximab can be persistently positive in 58% of patients [9]. Anti-DNA, C3 and C4 all were normal around and post the event of hemolysis. The time frame to clear lupus markers in case of Infliximab induced lupus reported by P Sarzi-Puttini is up to six months post withdrawal of the drug although other reports extend the period for one year or more [13](9). Furthermore, in lupus flaring the type of hemolysis mainly will be Coomb's positive autoimmune hemolysis while in our case scenario, Coomb's test was negative which indicate drug induced none immune hemolytic anemia by direct effect of Infliximab on RBC. Adding to that there was no arthritis or skin manifestation which was reported in almost all case reports that was published before as lupus-like syndrome induced post Infliximab infusion. Yet, a concomitant CD with other connective tissue disease or vasculitis can occur irrelevantly to the event that happened in our case scenario.

Our case presentation of hemolysis post second dose of Infliximab (Remsima) and delayed response to steroid therapy was pointing toward a non-immune mediated process. Retrieving the literature did not show a case of Infliximab induced hemolysis in a CD but was reported in UC. For that we are reporting our case as it is a rare presentation and can be fatal if not detected. This may mandate the need of close monitoring of patients with CD post Infliximab (Remsima) infusion for any hemolysis or other complications.

## 4 Conclusion

Drug induced Hemolysis is one of the complications that is not well-known post receiving Tumor necrosis factor Inhibitor (TNF) -α (Remsima - infliximab) infusion. Coomb's negative hemolytic anemia keeps in favor of non-immune drug induced hemolysis rather than other differential or causative factors in our case scenario. Although cross-reactivity is expected between one biological agent to another, in our case using Adalimumab as an alternative choice instead of Tumor necrosis factor Inhibitor (TNF) -α (Remsima - infliximab) did not cause hemolysis or any type of reaction.

## 5 Learning points

• Tumor necrosis factor Inhibitor (TNF)-α (Remsima) can induce hemolytic anemia in patients with Crohn's disease. Further study is required.
• Cross-reactivity is one of the major obstacles to manage the patient with this category, however we did not face the same risk.

## Ethical approval

This is case report paper, as per KFMMC-Dhahran ethics committee, no IRB is required only patient consent which already taken.

## Sources of funding

I declare that, there is no sponsors for our paper or funding.

## Author contribution

Rehab Y AL-Ansari: written the paper and diagnosed the event. Arwa AL Khuraim: collect the data. Leena Abdalla: written and review the paper. Hind Hamid: Writing in patient management part and first reviewer of the manuscript. Nawaf Zakary: review and correction of the paper.

## Trial registry number

Not applicable.

## Guarantor

Rehab Y AL-Ansari, Adult Hematology unit, Internal medicine Department, KFMMC, Dhahran 31,932, Kingdom of Saudi Arabia, Email: dr_rehab10000@hotmail.com, rehab@kfmmc.med.sa.

## Consent

Written informed consent for publication of their clinical details and/or clinical images was obtained from the patient. A copy of the written consent is available for review by the Editor-in-Chief of this journal on request.

## Declaration of competing interest

I declared that, no conflicts of interest to be report.
